# My 42-year Experience in Radiation Oncology

**DOI:** 10.14789/jmj.JMJ22-0025-R

**Published:** 2022-08-15

**Authors:** KEISUKE SASAI

**Affiliations:** 1 Department of Radiation Oncology, Juntendo University, Graduate School of Medicine, Tokyo, Japan; 1 Department of Radiation Oncology, Juntendo University, Graduate School of Medicine, Tokyo, Japan; 2 Department of Radiation Therapy, Misugikai Satou Hospital, Osaka, Japan; 2 Department of Radiation Therapy, Misugikai Satou Hospital, Osaka, Japan

**Keywords:** radiation therapy, radiation oncology, image-guided radiation therapy, intensity modulated radiation therapy, biological target volume

## Abstract

In the present review, I provide an overview of the development of radiation therapy and short history of the Department of Radiation Oncology, Juntendo University. I also emphasize the importance of radiation therapy as a major treatment modality for cancers.

Radiation therapy is a standard treatment for malignant tumors. It aims to deliver a sufficient radiation dose to a target volume to eradicate tumor cells or relieve symptoms of disease. Therapy can achieve good results in many types of cancers. Although radiation therapy sometimes causes undesirable adverse events, it is generally less invasive than other treatment modalities and does not alter the shape and function of healthy organs. When the author joined this field in 1981, radiation therapy techniques were highly primitive; however, during the past 42 years, treatment has advanced rapidly with the development of computer science, mechanical techniques and instrumentation. Currently, patients can be treated with precise radiation techniques, including intensity-modulated radiation therapy, image-guided radiation therapy, stereotactic irradiation, and brachytherapy. We also introduced a new treatment planning system that uses not only anatomical but also metabolic imaging, which permits correct delineation of the target volume. Therefore, it is crucial to stay up to date with advances and developments in rapidly emerging technologies to maintain high-quality treatment. The Department of Radiation Oncology at Juntendo University (Tokyo, Japan) is still small; however, it is gradually expanding and conducting research in both clinical and basic fields. It is the author's hope that many young investigators will join this field in the future.

## Introduction

Radiation therapy is a standard treatment for malignant diseases. It is a treatment modality that aims to deliver a sufficient radiation dose to a target volume to eradicate tumor cells or relieve symptoms of disease.

In the United States, approximately one-half of patients with cancers undergo radiation therapy; however, only one-quarter of such patients are irradiated in Japan^1)^. There are several reasons for this low frequency of radiation therapy. For example, the number of cancers that are not candidates for radiation therapy, such as gastric malignancy, are higher than those in the United States or in European countries. Additionally, as a result of the destructive atomic bombings of Hiroshima and Nagasaki (Japan), and the Fukushima nuclear catastrophe, many Japanese generally fear radiation and radiotherapy.

In the present review, I provide an overview of the development of radiation therapy and short history of the Department of Radiation Oncology, Juntendo University (Tokyo, Japan). I also emphasize the importance of radiation therapy as a major treatment modality for cancers.

## Developments in radiation therapy since 1981

When I joined this field in 1981, radiation therapy techniques were highly primitive. Virtually all institutions in Japan were equipped only with low-energy photon sources, such as cobalt-60 machines or low-energy medical linear accelerators (Linacs). Treatment was usually performed using a simple technique, such as two opposing anterior-posterior and posterior-anterior ports. The field was trimmed using one or two monoblocs fabricated from thick heavy metals. The radiation treatment field was determined using an X-ray simulator, which is a type of X-ray fluoroscopy specifically designed for radiation therapy treatment planning, and it has the same geometric arrangement as the treatment device. The field was determined based on anatomical landmarks, such as bones, which are visualized using X-rays. For example, radiation fields for uterine cervical cancer were determined at the upper end, between the 4th and 5th lumbar vertebrae, the lateral margin at 1.5 cm lateral to the inner margin of the iliac bone, and the lower margin at the level of the caudal end of the obturate foramen. However, some institutions did not have an X-ray simulator; as such, they had to use fluoroscopy dedicated to diagnostic use or simple X-ray photography. Low-energy photons cannot sufficiently penetrate to deep-seated areas of disease because they rapidly lose their energy along their track in the human body. Therefore, an extremely high dose was administered to the skin to treat deep-seated tumors, which sometimes caused severe side effects. The aforementioned factors, therefore, limited cure to only diseases located in superficial regions or easily approachable tumors, such as uterine cervical cancers in the early 1980s. As such, it was generally believed that radiation therapy was not a curative but a palliative method.

**Figure 1:**
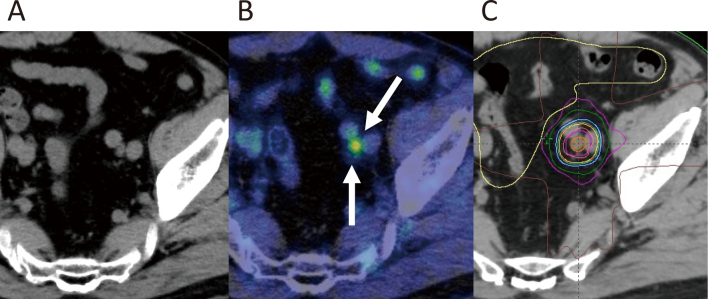
A male patient with prostate cancer underwent salvage radiation therapy for biochemical recurrence after surgical resection. However, his prostate-specific antigen levels gradually increased after five years. Computed tomography (CT) could not detect any recurrent lesions (A); however, ^18^F- fluorodeoxyglucose positron emission tomography combined with CT clearly revealed suspected lymph node disease (B, indicated by white arrows). This lesion was treated using stereotactic radiation therapy using a total dose of 50 Gy in 10 fractions. Dose distributions of the treatments (C). Prostate-specific antigen levels dramatically decreased after treatment.

However, rapid advances in computer science and mechanical technologies have led to a revolution in the field of radiation oncology. Linacs with ultra-high-energy X-rays, which can easily reach deep-seated lesions, are now commercially available. In the final decade of the 20th century, many new techniques emerged. If sufficient radiation doses could be delivered to the target volume, desirable performance in treatment was achieved. Stereotactic radiosurgery uses three-dimensional images and focuses multidirectional beams on a small target. The treatment was first applied to intracranial lesions, then gradually extended to extracranial diseases (Figure 1) and has yielded a very high frequency of disease eradication. From Juntendo University, Naoi and colleagues were pioneers in this field in Japan and published high-quality reports^2, 3)^.

**Figure 2:**
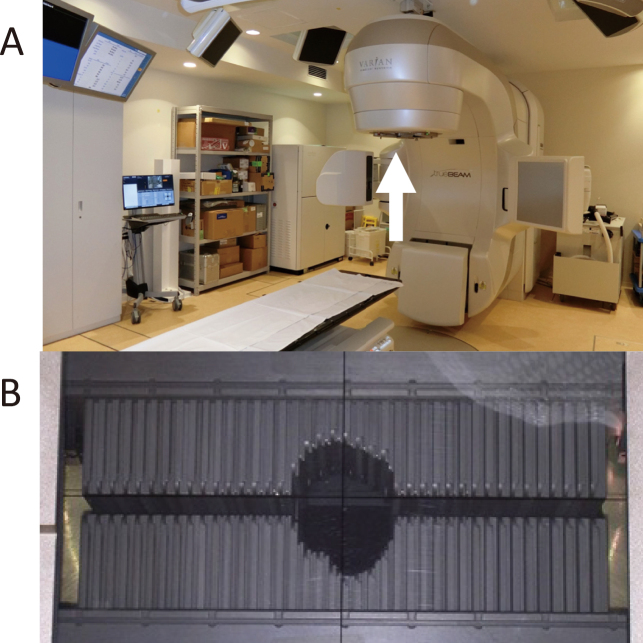
A medical linear accelerator (Linac) at the Juntendo University Hospital (Tokyo, Japan) (A) and multileaf collimator (B) placed at the aperture of the device, indicated by the white arrow.

**Figure 3:**
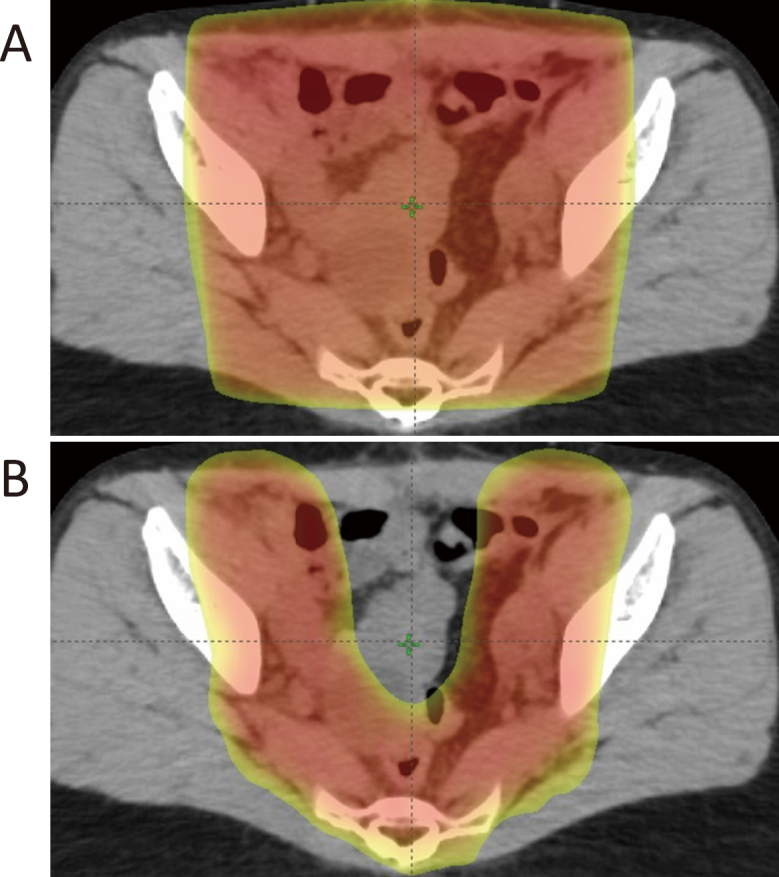
Dose distributions in conventional conformal radiation therapy (A) and intensity modulation radiotherapy (B).

Takahashi and colleagues proposed “conformation radiation therapy” in the 1960s^4, 5)^. This is a type of rotational radiation therapy, in which the tumor(s) is irradiated in a 360°direction, and the beams are trimmed to conform to the shape of the target volume during irradiation. However, the technique was not very popular until the 1990s because it was very complicated and there was no way to obtain trans-axial images of the body except by using Takahashi's rotation tomograms. In the 1990s, major instrument manufacturers equipped their Linacs with a multi-leaf collimator (Figure 2), which can easily shape the radiation field to conform to the target. Computed tomography (CT), which was introduced in the early 1970s, has also advanced to provide sufficient image quality for treatment planning. Since then, a new technique, known as “conformal radiation therapy”, in which a target is irradiated by conformal beams from several fixed directions, has emerged and is widely used. At the turn of the new millennium, a more sophisticated treatment technique, known as intensity-modulated radiation therapy (IMRT), has been introduced in this field^6, 7)^, with developments in this technology advancing virtually every year. It can be used to treat patients using an acceptable dose distribution (Figure 3).

**Figure 4:**
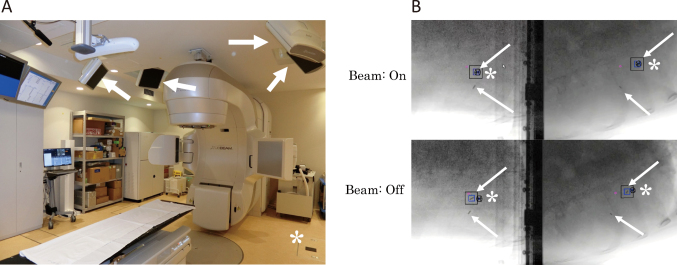
(A) The tracking system on a medical linear accelerator (Linac). The white arrows indicate X-ray detecting boards and the asterisk indicates one of four X-ray sources placed under the floor. (B) We usually use two sets of X-ray systems to tract a gold fiducial marker placed near the target volume. While the marker is out of position, the Linac beam is off. If the marker moves into the predefined position on images from both directions, the radiation beam is on.

Another advance in treatment is the introduction of image-guided radiation therapy (IGRT)^8)^. IMRT has a steep fall-off of the radiation dose at the edge of the target volume. If the position of the target volume differs in a radiation session from the planning CT, the volume receives a lower dose than the plan prescribes. To overcome this problem, the position of the target is monitored before or during each treatment session using imaging modalities such as CT, magnetic resonance imaging (MRI), and/or ultrasound. Furthermore, it is possible to detect the movement of the target during irradiation. At Juntendo University Hospital in Hongo, tumor movement was tracked during a treatment session using the SyncTrax system (Shimadzu, Kyoto, Japan) (Figure 4), a real tract radiation system^9)^. It is combined with stereotactic radiation therapy to mainly treat lung or liver cancers. This technique, however, has drawbacks, including exposure to X-rays and the visualization of only the fiducial markers inserted near the target volume. A newly emerged MRI-guided treatment technique can detect the movement of the target itself without any harmful effects^10)^, and may be a future direction of research and therapeutics.

In 2000, Ling et al. proposed a concept known as “biological target volume”^11)^. As mentioned above, multiple modalities can be used to precisely irradiate the target volume. However, defining the target volume, which is usually based on anatomical images, remains a problem. Ling et al. proposed the use of biological and mechanistic data to delineate target volumes. Biological images broadly include metabolic, biochemical, physiological, functional, molecular, genotypic, and phenotypic. Although positron emission tomography (PET) using ^18^F- fluorodeoxyglucose (FDG) is available for this purpose at Juntendo (Figure 1), other imaging modalities, such as ^18^F-misonidazole PET for hypoxic cells, have been tested at other institutions^12, 13)^. With advances in diagnostic and imaging modalities, functional imaging is anticipated to be introduced in this field in the future.

Lack of qualified personnel in this field is a major issue in Japan. There were only 899 certified radiation oncologists (ROs), 1213.9 full-time equivalent (FTE) ROs, and 295.7 FTE medical physicists, despite 846 institutions treating patients using radiotherapy in 2015^1)^. Juntendo also contends with this problem, and will be addressed later.

## Radiation therapy as a standard treatment for cancer

As shown in Table 1, radiation therapy can achieve good treatment results. Although it sometimes causes undesirable adverse events, it is generally less invasive than other modalities, and can preserve the shapes and functions of healthy organs. Therefore, it is regarded to be standard treatment for many types of malignant diseases and appears in domestic and international treatment guidelines for cancers. The therapy can be used not only as monotherapy, but can also be combined with other methods, including surgery, chemotherapy, and/or immunotherapy.

**Table 1:** Radiation therapy results for representative diseases at Juntendo University Hospital

Disease		Local control (%)	5-year (%)	7-year (%)	10-year (%)
Prostatic cancer	Low risk		100*	100*	100*
	Intermediate risk		95.1*	92.0*	89.2*
	High risk		96.1*	93.2*	85.7*
	Salvage radiation therapy		83.6*	76.7*	
Breast cancer	Conventional fractionation		98.7**		95.9**
	Accelerated fractionation		98.3**		95.3**
Uterine cervical cancer ***	Stage I		100***		
	Stage II		84***		
	Stage III		78***		
	Stage IVA		40***		
SRT for lung cancer		96			

*Biochemical relapse free rate, **the ipsilateral breast tumor control rate, Yoshida-Ichikawa Y et al. Breast Cancer 2021 Jan; 28(1): 92-98 ***Overall survival, SRT: stereotactic radiation therapy

## Radiation therapy at Juntendo University

When I was appointed to Juntendo University in 2000, the Radiation Oncology Division was a small part of the Department of Radiology. There was only one other RO with the exception of myself, although the individual was young and uncertified. Juntendo University had only two old-type Linacs (one at Juntendo University Hospital and another at Urayasu Hospital, Chiba, Japan).

The term “radiology” does not necessarily refer to radiation oncology (therapy) but refers to diagnostic radiology in the United States and major European countries. Because cancer is a leading cause of death, the Japanese government created the “Basic Plan to Promote Cancer Control Programs” based on the Cancer Control Act. One of the major policies is to promote radiation therapy, with the government encouraging each medical school to establish a radiation oncology department. In 2013, Juntendo University also established the Department of Radiation Oncology, and I was appointed to be the first Chair. Juntendo Hospital is now equipped with three cutting-edge Linacs and a remote after-loading brachytherapy system. These facilities permit the treatment of virtually all cancer types that are suitable candidates for radiation therapy. Table 1 summarizes the results of radiation therapy for major diseases in our department^14)^. Generally, these values were better than expected. Other affiliated hospitals have also been equipped with new instruments, including Shizuoka Hospital (Shizuoka, Japan), with one; Urayasu Hospital, with two, and Nerima Hospital (Tokyo, Japan), with one (Table 2). Tables 2 and 3 summarize the changes in the radiation therapy facilities in the Juntendo University group and the number of patients treated at Juntendo Hospital, Hongo. Although there are few members in the Department of Radiation Oncology, the number has gradually increased to 11 ROs and 3 physicists.

**Table 2:** Changes of the radiation therapy facilities in Juntendo University from 2000 to 2021

	2000	2021
Linear accelerator	2 (H:1, U:1)	7 (H: 3, S:1, U:2, N:1)
Remote after loading system	1 （U:1）	1 （H:1）
Radiation Oncologist	2 (H:2)	11 (H:6, S:2, U:1, N:2)
Medical physicist	0	3 (+2)* (H:2 (+1)*, U:1, N(1)*)

H: Juntendo Hospital, S: Shizuoka Hospital, U: Urayasu Hospital, N: Nerima Hospital, * numbers in parenthesis mean medical physicists at the faculty of health science

**Table 3:** Changes of numbers of patients who received radiation therapy at Juntendo Hospital, Hongo, from 2000 to 2020

	2000	2020
Patients who received radiation therapy	450	1003
IMRT	0	271
SRT	32	55
brachytherapy	0	47

IMRT: intensity modulated radiation therapy including VMAT (volumetric-modulated arc therapy) SRT: stereotactic radiation therapy (including stereotactic radiosurgery)

## Research at the Department of Radiation Oncology

Research themes at the Department of Radiation Oncology include both the basic and clinical fields, which is very similar to themes in other departments. Basic research includes both medical physics and radiation biology. Although my majors were clinical and radiation biological research, the lack of personnel was limited to the clinical and medical physics themes at Juntendo. During these years, the department published only a few articles in English; however, this number is now increasing as the number of members in our department has increased. Among these publications, Akamatsu et al. reported a close relationship between the prognosis of patients with esophageal squamous cell carcinoma and the expression of c-erbB-2 in tumor tissue^15)^. Kunogi et al. predicted the radiosensitivity of tumor cells by simultaneously detecting histone H2AX phosphorylation and apoptosis^16)^. Recently, we reported that patients who underwent radiation therapy for cranial diseases experienced unusual visual and olfactory sensations^17, 18)^. Among them, one woman who underwent resection of the olfactory bulb and epithelium reported a pungent smell during the radiation session^19)^. This phenomenon suggests that the central nervous system can detect X-rays, even in humans.

## Conclusions

Radiation therapy has evolved from very primitive techniques to a highly sophisticated and precise level during the past 40 years, along with advances and developments in instrumentation and techniques. It is crucial to stay up to date with these developments to maintain high-quality treatment. The department of radiation oncology is currently small but has gradually expanded year by year. It is my hope that many young investigators will join this field in the future.

## Funding

No funding was received.

## Author contributions

KS contributed to the conception, drafting the manuscript, and preparation of figures and tables.

## Conflicts of interest statement

The Author declares that there are no conflicts of interest.

## References

[1] Numasaki H, Nakada Y, Okuda Y, Ohba H, Teshima T, Ogawa K: Japanese structure survey of radiation oncology in 2015. J Radiat Res, 2022; 63: 230-246. 10.1093/jrr/rrab129 PMC8944304 35137180

[2] Naoi Y, Cho N, Miyauchi T, Iizuka Y, Maehara T, Katayama H: Usefulness and problems of stereotactic radiosurgery using a linear accelerator. Radiat Med, 1996; 14: 215-219. 8916267

[3] Naoi Y, Maehara T, Cho N, Katayama H: Stereotactic radiosurgery for brain metastases using a linac system: evaluation of initial local response by imaging. Radiat Med, 1999; 17: 311-315. 10510905

[4] Kitabatake T, Takahashi S: Conformation radiotherapy by means of a 6 MeV linear accelerator. Tohoku J Exp Med, 1968; 94: 37-43. 10.1620/tjem.94.37 5655293

[5] Takahashi S: Conformation radiotherapy. Rotation techniques as applied to radiography and radiotherapy of cancer. Acta Radiol Diagn (Stockh). 1965: Suppl 242: 1+. 5879987

[6] Purdy JA: Advances in three-dimensional treatment planning and conformal dose delivery. Semin Oncol, 1997; 24: 655-671. 9422262

[7] Zelefsky MJ, Leibel SA, Kutcher GJ, Fuks Z: Three-dimensional conformal radiotherapy and dose escalation: where do we stand? Semin Radiat Oncol, 1998; 8: 107-114. 10.1016/s1053-4296(98)80006-4 9516591

[8] Mackie TR, Kapatoes J, Ruchala K, et al: Image guidance for precise conformal radiotherapy. Int J Radiat Oncol Biol Phys, 2003; 56: 89-105. 10.1016/s0360-3016(03)00090-7 12694827

[9] Harada T, Shirato H, Ogura S, et al: Real-time tumor-tracking radiation therapy for lung carcinoma by the aid of insertion of a gold marker using bronchofiberscopy. Cancer, 2002; 95: 1720-1727. 10.1002/cncr.10856 12365020

[10] Sritharan K, Tree A: MR-guided radiotherapy for prostate cancer: state of the art and future perspectives. Br J Radiol, 2022; 95: 20210800. 35073158 10.1259/bjr.20210800 PMC8978250

[11] Ling CC, Humm J, Larson S, et al: Towards multidimensional radiotherapy (MD-CRT): biological imaging and biological conformality. Int J Radiat Oncol Biol Phys, 2000; 47: 551-560. 10.1016/s0360-3016(00)00467-3 10837935

[12] Okamoto S, Shiga T, Yasuda K, et al: The reoxygenation of hypoxia and the reduction of glucose metabolism in head and neck cancer by fractionated radiotherapy with intensity-modulated radiation therapy. Eur J Nucl Med Mol Imaging, 2016; 43: 2147-2154. 10.1007/s00259-016-3431-4 27251644

[13] Tamaki N, Hirata K: Tumor hypoxia: a new PET imaging biomarker in clinical oncology. Int J Clin Oncol, 2016; 21: 619-625. 10.1007/s10147-015-0920-6 26577447

[14] Yoshida-Ichikawa Y, Horimoto Y, Shikama N, et al: Ipsilateral breast tumor control following hypofractionated and conventional fractionated whole-breast irradiation for early breast cancer: a long-term follow-up. Breast Cancer, 2021; 28: 92-98. 10.1007/s12282-020-01134-8 32719997

[15] Akamatsu M, Matsumoto T, Oka K, et al: c-erbB-2 oncoprotein expression related to chemoradioresistance in esophageal squamous cell carcinoma. Int J Radiat Oncol Biol Phys, 2003; 57: 1323-1327. 10.1016/s0360-3016(03)00782-x 14630269

[16] Kunogi H, Sakanishi T, Sueyoshi N, Sasai K: Prediction of radiosensitivity using phosphorylation of histone H2AX and apoptosis in human tumor cell lines. Int J Radiat Biol, 2014; 90: 587-593. 10.3109/09553002.2014.907518 24708165

[17] Obinata M, Yamada K, Sasai K: Unusual olfactory perception during radiation sessions for primary brain tumors: a retrospective study. J Radiat Res, 2019; 60: 812-817. 10.1093/jrr/rrz060 PMC6873622 31553454

[18] Hara N, Isobe A, Yamada K, et al: Unusual visual and olfactory perceptions during radiotherapy sessions: an investigation of the organs responsible. J Radiat Res, 2021; 62: 718-725. 10.1093/jrr/rrab033 PMC8273799 33912958

[19] Kosugi Y, Hara N, Isobe A, Matsumoto F, Sasai K: Detection of ionising radiation by the CNS: a case report. Lancet Neurol, 2022; 21: 311-312. 10.1016/S1474-4422(22)00076-X PMC9761318 35305333

